# Biological markers: maintaining standards

**DOI:** 10.1054/bjoc.2000.1158

**Published:** 2000-04-27

**Authors:** Written on behalf of the Biomarkers Ad-hoc Group of the United Kingdom Coordinating Committee on Cancer Research


British Journal of Cancer (2000) 82(10), 1627–1628
© 2000 Cancer Research Campaign
DOI: 10.1054/ bjoc.2000.1158, available online at http://www.idealibrary.com on IDEAL®

### Biological markers: maintaining standards

**Written on behalf of the Biomarkers Ad-hoc Group of the United Kingdom Coordinating Committee on Cancer Research**

The potential value of biological markers in the management of cancer has been emphasized in several recent articles (Dowsett, 1998; Ad-hoc Biomarkers Group of UKCCCR, 2000). The focusing of cancer services within the UK and the proposals from the EORTC and others that any new clinical trials should include biomarker studies should mean that more centres will be undertaking biomarker analyses. Whilst the wider use of markers is to be applauded it does bring with it various potential problems that must be addressed, particularly in relation to methodology, quantification and defining cut-off points.

Biological markers can be determined in urine, serum and tissues. For urine and serum, a form of immunoassay is used, which allows quantification. However, for the markers to be of any use they have to be sensitive and specific, tests have to be reliable and the person using it has to be aware of any problems. For example, measurement of serum carcinoembryonic antigen (CEA) can be of value for monitoring colorectal cancer, but there are the following problems: smokers have higher circulating CEA concentrations than non-smokers; serum CEA can be elevated in a variety of acute and chronic inflammatory conditions; CEA can be elevated in cancers other than colorectal cancer. Although an individual CEA test kit gives comparable results, different CEA test methods do not give equivalent CEA values for individual samples, so the same test method should be used for a given patient (American Society of Clinical Oncology, 1999). If serological markers are to be used to monitor cancer progression or response to therapy, the test (and the laboratory) has to give reproducible, reliable results on repeated measures (Helzlsouer, 1994) and within defined limits in an external quality assurance (QA) system.

Determination of biological markers in tissues has more scope and there have been important changes that have occurred over the past 25 years. Immunoassays and saturation binding assays using homogenates of tissue, which had to be frozen promptly after removal, have been the main methods used. These have the drawback that tumours have to be obtained fresh, tissue snap-frozen, stored correctly and cytosols prepared in defined buffers, which limits their availability. Their advantage is that a numerical result can be obtained, e.g. Scatchard analysis of the dextran-coated charcoal radioactive ligand binding assay for oestrogen receptor (ER) is reported as femtomoles of oestradiol bound per mg of cytosol protein. The results can be divided into positive and negative on the basis of the clinical cut-off value of 10 fmol $mg^{-1}$ protein, and different sub-groups evaluated (Hawkins et al, 1980). However, problems can arise with quantification, as shown by the wide coefficient of variations found for enzyme-linked immunosorbent assay (ELISA) analysis of urokinase-type plasminogen activator in breast cancer cytosols (Sweep et al, 1998). Data from trans-European studies has shown that for multicentre studies the same ELISA kit should be used, that external QA is mandatory and standardization of protein assays is imperative.

The introduction of antibodies directed against biological markers that can be applied to fixed tissue and improvements in antigen retrieval techniques (e.g. pressure cookers!) has meant that many laboratories have changed to immunohistochemical methods and also that many more laboratories are determining biological markers. This is a positive development but it is very important that there is standardization of all aspects of the assessment. Critical areas include adequate, prompt fixation (analogous with freezing tissues properly) so that there is even penetration of the whole tissue, the use of a carefully evaluated antibody, controlled antigen retrieval and a sensitive immunohistochemical detection method. Positive and negative controls are critical for each batch of staining. There have been issues as to whether there is a deterioration in immunoreactivity of certain antigens, e.g. p53, with storage of paraffin sections. While it is important to have in-house checks about the latter, probably more important is the use of optimally prepared tissues and a sensitive reliable method (Cooper et al, 1998).

Any assay that is to be used clinically must have good QA procedures. These exist for cytosol assays, e.g. ER, and there are excellent quality assurance schemes organized by the EORTC (Romain et al, 1995). An essential feature of these schemes is that they use a stable, easily distributed standard material and that quantitative elements of the assay are assessed. There are QA schemes for checking immunohistochemical staining (e.g. UK NEQAS ICC), to try and standardize methodology between laboratories. The problem comes with the quantification of immunohistochemistry and how it should be standardized. Defining clinically relevant cut-off values can be more difficult. ER again provides a good example; a variety of scoring systems have been used but they are subjective and semi-quantitative. Cut-off values for positive and negative, or response/non-response to endocrine treatment may vary depending on whether it is for adjuvant use or for treating metastatic disease. If, as for the former, the cut-off levels are low (Elledge and Osborne, 1997) then the methods and interpretation have to have a high sensitivity. QA of interpretation is just as important as that for the methods (Barnes et al, 1998). For

Correspondence to: R Leake, Division of Biochemistry and Molecular Biology, Davidson Building, Institute of Biomedical & Life Sciences, University of Glasgow, Glasgow G12 8QQ, UK

Membership of the Biomarkers Group Comprises: Professor Robin Leake (Biochemistry, Glasgow University – Chairman), Dr Liz Anderson (Clinical Research, Christie Hospital, Manchester), Professor Mitch Dowsett (Endocrinology, Royal Marsden Hospital, London), Dr Ray Iles (St Bartholomew's Hospital, London), Professor Rob Nicholson, Tenovus Unit, Pharmacology, Cardiff), Professor John Robertson (Surgery, Nottingham), Dr Rosemary Walker (Pathology, Leicester)

small samples and when normal and tumour are admixed, immunohistochemistry does have advantages.

The future is certainly brighter for biological markers but could be dimmed unless important standards are maintained.

### REFERENCES

Ad-hoc Biomarkers Group of UKCCCR (2000) Can biological markers improve cancer patient management? *Br J Cancer* **82**: (in press)

American Society of Clinical Oncology (1994) Clinical practice guidelines for the use of tumor markers in breast and colorectal cancer. *J Clin Oncol* **14**: 2843–2877

Barnes DM, Millis RR, Beex LVAM, Thorpe SM and Leake RE (1998) Increased use of immunohistochemistry for oestrogen receptor measurement in mammary carcinoma: the need for quality assurance. *Eur J Cancer* **34**: 1677–1682

Cooper LS, Gillett CE, Hanby AM and Barnes DM (1998) P53 immunoreactivity in breast cancer tissue does not deteriorate in stored paraffin sections. *Breast* **7**: 260–264

Dowsett M (1998) Improved prognosis for biomarkers in breast cancer. *Lancet* **351**: 1753–1754

Elledge RM and Osborne CK (1997) Oestrogen receptors and breast cancer. *Br Med J* **314**: 2843–1844

Hawkins RA, Roberts MM and Forrest APM (1980) Oestrogen receptors and breast cancer: current status. *Br J Surg* **67**: 152–169

Helzlsouer KJ (1994) Serological markers of cancer and their applications in clinical trials. *Cancer Res* **54**: 2011s–2014s

Romain S, Laine Bidrou C, Martin PM and Magdelenat H. (1995) EORTC receptor study group report: steroid receptor distribution in 47 892 breast cancers. A collaborative study of 7 European laboratories. *Eur J Cancer* **31A**: 411–417

Sweep CGJ, Guerts-Moespor J, Grebenschikov N, de Witte JH, Heuvel JJTM, Schmitt M, Dutty MJ, Janicke F, Kramer MD, Foekens JA, Brunner N, Brugal G, Pedersen AN and Benrald ThJ (1998) External quality assessment of trans-European multicentre antigen determinations (enzyme-linked immunoasorbant assay) of urokinase-type plasminogen activator (uPA) and its type 1 inhibitor (PAI-1) in human breast cancer extracts. *Br J Cancer* **78**: 1434–1441

*© 2000 Cancer Research Campaign*

## References

[PDF_00114] Barnes D. M., Millis R. R., Beex L. V., Thorpe S. M., Leake R. E. (1998). Increased use of immunohistochemistry for oestrogen receptor measurement in mammary carcinoma: the need for quality assurance.. Eur J Cancer.

[PDF_00120] Dowsett M. (1998). Improved prognosis for biomarkers in breast cancer.. Lancet.

[PDF_00122] Elledge R. M., Osborne C. K. (1997). Oestrogen receptors and breast cancer.. BMJ.

[PDF_00124] Hawkins R. A., Roberts M. M., Forrest A. P. (1980). Oestrogen receptors and breast cancer: current status.. Br J Surg.

[PDF_00126] Helzlsouer K. J. (1994). Serological markers of cancer and their applications in clinical trials.. Cancer Res.

[PDF_00128] Romain S., Lainé Bidron C., Martin P. M., Magdelenat H. (1995). Steroid receptor distribution in 47,892 breast cancers. A collaborative study of 7 European laboratories. The EORTC Receptor Study Group.. Eur J Cancer.

[PDF_00132] Sweep C. G., Geurts-Moespot J., Grebenschikov N., de Witte J. H., Heuvel J. J., Schmitt M., Duffy M. J., Jänicke F., Kramer M. D., Foekens J. A. (1998). External quality assessment of trans-European multicentre antigen determinations (enzyme-linked immunosorbent assay) of urokinase-type plasminogen activator (uPA) and its type 1 inhibitor (PAI-1) in human breast cancer tissue extracts.. Br J Cancer.

